# Effectiveness of an exercise-based prehabilitation program for patients awaiting surgery for lumbar spinal stenosis: a randomized clinical trial

**DOI:** 10.1038/s41598-021-90537-4

**Published:** 2021-05-26

**Authors:** Andrée-Anne Marchand, Mariève Houle, Julie O’Shaughnessy, Claude-Édouard Châtillon, Vincent Cantin, Martin Descarreaux

**Affiliations:** 1 Department of Chiropractic, Université du Québec à Trois-Rivières, 3351 Boul. des Forges, Trois-Rivières, QC G9A 5H7 Canada; 2 Department of Anatomy, Université du Québec à Trois-Rivières, Trois-Rivières, Canada; 3 Centre Intégré Universitaire de Santé et de Services Sociaux de la Mauricie-et-du-Centre-du-Québec, Trois-Rivières, Canada; 4 Division of Neurosurgery, Faculty of Medicine, University of Montreal, Montréal, Canada; 5 Department of Human Kinetics, Université du Québec à Trois-Rivières, Trois-Rivières, Canada

**Keywords:** Neurological manifestations, Spondyloarthritis, Musculoskeletal system

## Abstract

Lumbar spinal stenosis is the most common reason for spine surgery in older adults, but the effects of prehabilitation on perioperative outcomes among these patients have not been investigated. This study aims to evaluate the effectiveness of a preoperative exercise-based intervention program compared with usual care on the improvement of clinical status, physical capacities and postoperative recovery of patients awaiting surgery for lumbar spinal stenosis. Sixty-eight participants were randomised to receive either a 6-week supervised exercise-based prehabilitation program or hospital usual care. The outcomes included both clinical and physical measures. Data collection occurred at post-intervention, and 6 weeks, 3- and 6-months post-surgery. Significant but small improvements were found in favour of the experimental group at the post-intervention assessment for pain intensity, lumbar spinal stenosis-related disability, lumbar strength in flexion, low back extensor muscles endurance, total ambulation time, and sit to stand performance. A significant difference in favor of the intervention group was found starting at the 3-month postoperative follow-up for low back-related disability. No adverse events were reported. Exercise-based prehabilitation did not improve short-term postoperative recovery in patients with lumbar spinal stenosis.

## Introduction

As populations worldwide continue to grow older, increasing demand is to be expected on health care systems, including surgery-related resources. The metabolic response that results from surgical procedures is characterized by changes in body composition, loss of muscle function and strength, decreased vasomotor control, and sympathetic hyperactivity^1,2^. Considering that the extent of this response impacts postoperative recovery and long-term outcomes, attempts to minimize it have become paramount for the perioperative teams^3,4^.

The aging process is naturally associated with some degree of physical deconditioning^5,6^, which compromises the physiologic reserve required to better withstand the stress of surgery^7^. Furthermore, loss of physiologic reserve is aggravated by sedentary behavior that in turn contributes to negative consequences on functional independence^8^. Physical exercise, including resistance and aerobic training, is part of the solution to attenuate physical decline in older individuals^9,10^. As such, interventions aimed at augmenting patients’ physiological fitness prior to a surgical intervention to allow them to increase their metabolic reserve and retain a higher level of functional capacity over their entire perioperative trajectory, are known as prehabilitation^8,11^.

Most publications on prehabilitation pertain to life-threatening diseases and complex surgeries while the body of evidence in the context of elective spine surgery remains scarce^12–14^. In a recent systematic review, Janssen et al. reported on the effectiveness of prehabilitation in patients with degenerative disorders of the lumbar spine^15^. Based on data from twelve interventions, the authors concluded that prehabilitation has no effect when compared to usual care in patients undergoing lumbar spinal surgery. However, only one of the included studies (with unclear risk of bias) looked at exercise^16^, which was not enough to draw any conclusion regarding the effectiveness of exercise-based prehabilitation^15^.

Lumbar spinal stenosis (LSS) is one of the most frequent degenerative conditions in older-aged patients^17^ and represents the main reason for undergoing surgery in adults over the age of 65^18^. It is hallmarked by neurogenic claudication, causing high levels of disability, disrupting activities of daily leaving and leading to a more sedentary lifestyle^19^. With as little as 4% of patients meeting the Canadian recommendations for physical activity^20^ and considering that watchful-waiting is safe in this slowly progressing condition^21^, LSS may be best suited to study the effect of prehabilitation prior to spine surgery.

Therefore, the aim of the study was to assess the effectiveness of an active exercise-based prehabilitation programme compared to usual care in patients with LSS. It was hypothesized that patients in the intervention group would have greater preoperative functional capacities, which would lead to faster post-operative recovery, compared to the control group.

## Methods

### Study design

The study was a single-centre, parallel-group randomized controlled trial with an internal pilot component. We previously conducted a pilot study to test the intervention, the choice of outcome measures, and to gather preliminary data. Given that the intervention was not modified between the pilot and the main trial, the forty participants from the pilot study were included in the final analysis presented herein. The trial was conducted at the Université du Québec à Trois-Rivières (UQTR) research facility, Canada. Enrollment started in February 2015, with the last follow-up in February 2020. The trial protocol^22^ as well as the feasibility and pilot results^23^ have been published elsewhere. The study received ethical approval from the institutional review board of UQTR (CÉR-2014-008-00) and was registered in ClinicalTrials.gov (NCT02258672; October 7th, 2014). All methods were carried out in accordance with relevant guidelines and regulations. Informed written consent was obtained from each participant prior to data collection.

### Participants

We included individuals ≥ 18 years, diagnosed with degenerative LSS primarily of central origin (confirmed with matching clinical history and diagnostic imaging), awaiting surgery (minimally invasive or open approach) and able to provide written informed consent voluntarily. Exclusion criteria included presence of non-degenerative LSS, inflammatory arthritic conditions, vertebral instability requiring non-instrumental or instrumented fusion and altered cognitive capacities; individuals deemed ineligible by their treating neurosurgeon; and being unable to understand or express oneself in French. All patients were recruited at the Centre intégré universitaire de santé et de services sociaux de la Mauricie-et-du-Centre-du-Québec (Trois-Rivières’ regional hospital (Quebec, Canada)) in collaboration with the neurosurgery team. Neurosurgeons were responsible for identifying eligible patients during outpatient clinical encounters. Patients meeting inclusion criteria and interested in the study were asked for consent to be later contacted by a member of the research team.

### Interventions

All participants, regardless of group allocation, received the day prior to surgery, standardized written information on how to keep a good back posture when getting in or out of bed and when sitting down after the surgery. That is the usual care provided by the hospital staff for all patients undergoing back surgery.

Participants in the exercise group were offered individually supervised exercise sessions 3 times per week for 6 weeks, prior to their surgery. Training sessions took place at the *Université du Québec à Trois-Rivières* rehabilitation facility and were led by a certified kinesiologist. A typical training consisted of a 5-min warm-up (stationary cycling or walking on a treadmill based on participants’ preference), followed by 25 min of exercises with concentric or isometric phases that aimed to improve muscle and structures involved in walking capacities. Exercise intensity level was individually tailored to the participants’ capacity and progressively modified to provide increasing levels of difficulty. For a full description of the exercise intervention, see previous reports^22,23^. Adherence to the exercise program was documented in the kinesiologist logbook. For each exercise, recorded data include the number of sets, repetitions and levels of difficulty reached (1 being the lowest and 4 the highest level of difficulty), perceived effort and location, intensity, and character of discomfort if any. Participants in the control group were not discouraged from performing physical activity or exercise.

### Outcomes

Sociodemographic data were collected via a structured interview and by self-reported questionnaires at baseline, with a trial researcher available to clarify questions if needed.

Treatment effect was assessed using both clinical patient-reported outcome measures and objective physical tests. Clinical patient-reported outcome measures were collected at UQTR’s research facility at baseline, 6-week from baseline (post-intervention), and 6 weeks post-surgery, and by post at 3- and 6-month post-surgery. Physical outcome measures were collected at UQTR’s research facility at baseline, after 6 weeks prehabilitation intervention, and 6 weeks post-surgery.

Primary outcome measures were current low back and leg pain intensity (11-point Numerical Rating Scale)^24^, and low back-related disability (Oswestry Disability Index)^25^.

Secondary outcome measures included quality of life (EuroQol-5D)^26^, fear avoidance behavior (Tampa Scale of Kinesiophobia)^27^, level of anxiety and depression (Beck Disability Index)^28^, patient perception of treatment effect (7-point scale Patient Global Impression of Change^29^—measured at the post-intervention assessment only), lumbar extensor muscles endurance (modified Sorensen test)^30^, trunk flexor and extensor muscle strength (isometric contraction)^31^, knees extensor muscle strength (isometric contraction)^32^, active lumbar ranges of motion^33^, and walking abilities (time to first symptoms and total ambulation time). Lastly, perioperative data including blood loss, length of surgery, surgical technique used, intraoperative complications, and length of hospital stay were documented as potential explanatory factors of between group differences in recovery. The study protocol provides further information about the selected outcomes^22^.

Based on results from the pilot study^23^, physical tests better reflecting patients’ activities of daily living were deemed necessary to capture functional capacities as oppose to physiological changes to exercise. As such, in the main trial, we included the 30 s sit-to-stand^34^ and the timed up and go^35^ tests that allowed for the measurement of progress regarding balance, sit to stand, and short-distance walking capacities. In addition, in response to the participants comment that the ODI did not completely capture their daily challenges we also added the French Swiss Spinal Stenosis questionnaire^36^ to measure LSS-related disability. These newly collected outcomes are available for the latest recruited participants only (15 and 13 from the intervention and control group respectively).

### Sample size

The sample size calculation for the main trial was conducted using the pilot study’s means and standard deviations for leg pain intensity^23^ (measured after the 6-week prehabilitation intervention), assuming a one-tailed test and considering a significance level of p = 0.01, a power of 90%, and a 20% attrition rate. An estimated 36 patients per treatment arm were required to detect a significant between-group difference.

### Randomization and blinding

Randomisation and minimization were performed after the baseline assessment using a computer random number generator, prepared by a research assistant not involved in the study process. Allocation concealment was ensured using sequentially numbered, opaque and sealed envelopes. The envelopes were opened in front of the participants by the main investigator after enrollment. Participants were not blinded to intervention allocation, but to prevent cross-contamination between groups content of exercise sessions was known only to those in the intervention group. Further details about randomisation, minimization and blinding are published elsewhere^22,23^.

### Statistical methods

For between-group comparisons of demographic and perioperative data, the independent Student t test for continuous variables and the chi-square test for categorical variables were used. Mixed model ANOVAs were used for group comparison over time and Bonferroni post hoc tests were conducted whenever necessary. Based on observations from the pilot study during which a significant effect of surgery was observed for primary clinical outcomes^23^, the analyses were first conducted using the baseline and post-intervention data, and then the post-intervention and follow-ups data together. Whenever baseline variables did not follow normal distribution using the Shapiro–Wilk test, appropriate transformations were applied in order to conduct parametric statistics. Analyses of primary and secondary outcomes were conducted according to the intention-to-treat principle with participants analyzed according to randomly assigned treatment group irrespective of compliance. Missing data (mean number = 19.1% per table) were replaced using multiple imputation regression modeling methods and an aggregate of 1000 imputed data sets was used to conduct the analysis of variance. All analyses were conducted in SPSS Statistics version 25.0. (IBM, Armonk, NY). The level of significance was set to 0.05.

## Results

### Recruitment

**Figure 1:**
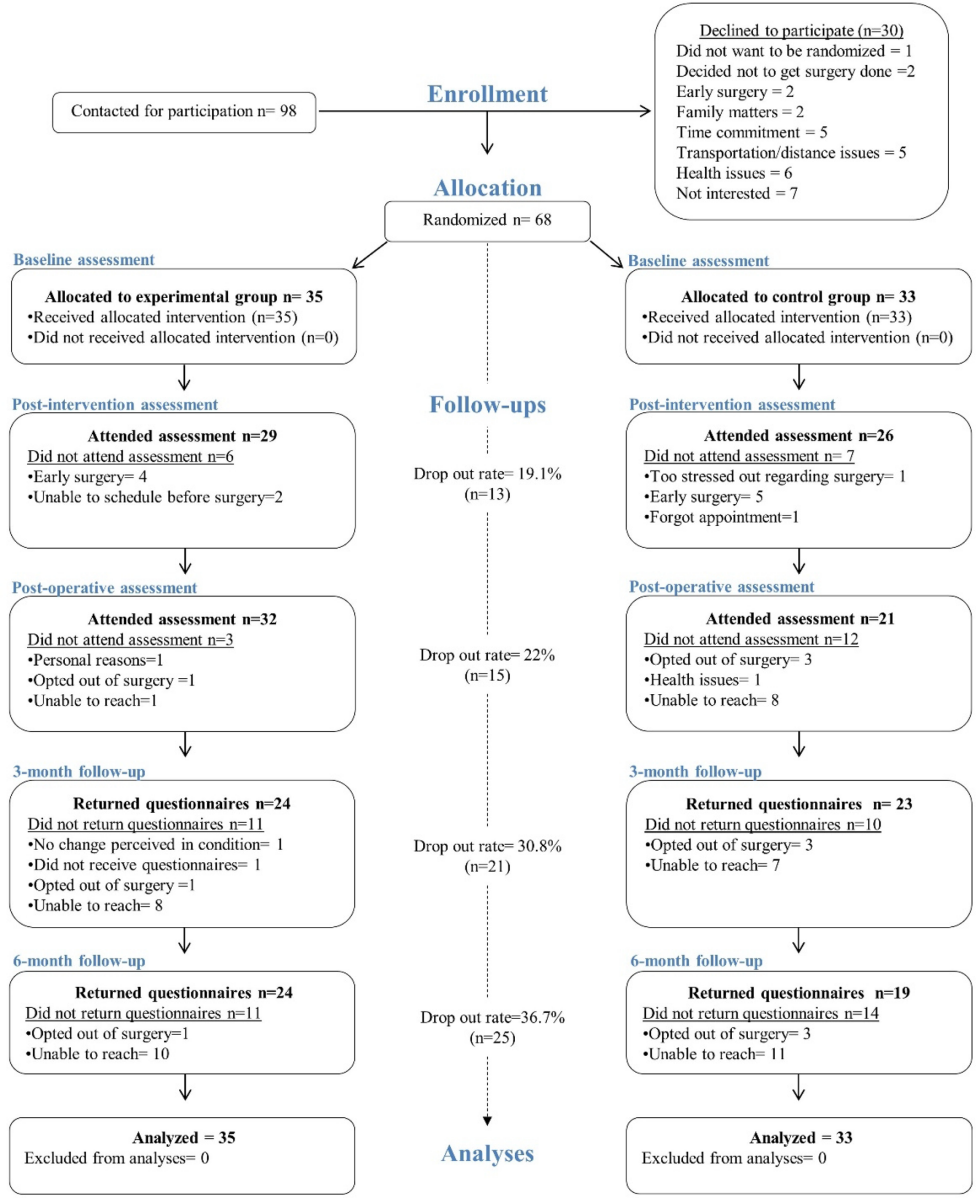
CONSORT flowchart of the randomized controlled trial.

Between February 2015 and June 2019, a total of 98 eligible patients were contacted, of whom 68 agreed to participate and were randomly assigned to the intervention (n = 35) or control group (n = 33). Due to the long follow-up period and a much lower patient load during summer months for the neurosurgeons, it was decided to stop the recruitment prematurely, with 94% of the recruitment goal achieved. Figure 1 presents participants flow in the study along with reasons for non-participation and attrition.

### Baseline data

There was no significant difference between the groups with respect to baseline characteristics except for age which was lower (p = 0.01) in the intervention group. Table 1 presents the baseline characteristics for all participants.

**Table 1:** Participants’ baseline characteristics.

	Intervention (N = 35) Mean ± SD	Control (N = 33) Mean ± SD	*p*
**Demographics**			
Age—years	66.2 ± 9.6	71.6 ± 7.6	0.01*
Gender, female—n (%)	14 (40)	14 (42)	0.83
BMI	29.0 ± 5.2	28.9 ± 5.2	0.90
Weight—kg	81.3 ± 16.5	82.7 ± 19.1	0.74
Height—cm	169.9 ± 11.7	166.2 ± 10.0	0.16
Minimisation criteria			
Diabetes—n (%)	4 (11)	7 (21)	0.27
Positive EMG findings—n (%)	7 (20)	3 (9)	0.20
ODI score ≥ 41%—n (%)	14 (40)	9 (27)	0.26
Smoker—n (%)	2 (5)	1 (3)	0.59
Employment situation—n (%)			0.39
Currently working	3 (8.6)	5 (15.2)	
Sick leave or retired due to pain	11 (31.4)	5 (15.2)	
Retired unrelated to pain	21(60)	23 (69.6)	
Work satisfaction—/100^a^	83.8 + 7.5	86.2 ± 10.9	0.49
Operated vertebral segment(s)			
1 level	18 (53%)	16 (53%)	0.97
2 levels	8 (24%)	8 (27%)	0.77
3 levels	7 (20%)	6 (20%)	0.95
4 levels	1 (3%)	0 (0%)	–
**Primary outcomes**			
Leg pain intensity—/10	7.4 ± 1.8	6.7 ± 2.3	0.16
**Secondary outcomes**			
Clinical outcomes			
Back pain intensity—/10	5.3 ± 3.0	5.5 ± 2.7	0.77
Leg pain dominant—n (%)	27 (77)	22 (66)	0.33
Weekly days with pain—(/7)	6.9 ± 0.1	6.9 ± 0.3	0.62
Back disability—/100	37.2 ± 16.4	37.4 ± 11.9	0.93
Kinesiophobia—/68	47.4 ± 8.0	45.2 ± 7.7	0.24
Depression—/63	4.4 ± 5.0	4.7 ± 4.1	0.79
Physical outcomes			
Lumbar active ROMs—degrees			
Flexion	66.0 ± 25.1	67.2 ± 20.8	0.84
Extension	14.4 ± 7.0	14.4 ± 5.4	0.99
Left lateral flexion	13.0 ± 7.3	12.0 ± 6.1	0.58
Right lateral flexion	13.8 ± 7.9	13.7 ± 6.8	0.97
Trunk muscles strength—N m			
Flexion	46.2 ± 23.4	44.1 ± 28.1	0.66
Extension	33.2 ± 28.8	28.4 ± 24.2	0.47
Right knee extensor strength—lbs	62.1 ± 31.7	60.7 ± 28.6	0.84
Left knee extensor strength—lbs	72.6 ± 27.3	75.6 ± 26.9	0.77
Lumbar extensor endurance—s	45.2 ± 59.9	38.7 ± 49.2	0.64
Walking capacities—s			
Time to 1st symptoms	116.8 ± 87.4	97.4 ± 93.4	0.38
Total ambulation time	196.7 ± 94.0	190.0 ± 139.1	0.81
**Newly added outcomes**^**b**^			
LSS disability—(/55)	39.5 ± 4.2	38.0 ± 4.2	0.40
Sit to stand—repetitions	9.6 ± 3.8	9.0 ± 2.7	0.65
Get up and go—s	8.3 ± 1.5	9.8 ± 3.8	0.23

*EMG* electromyography, *ODI* Oswestry disability index, *LSS* lumbar spinal stenosis.

*Statistically significant difference between groups.

^a^Measured using the Minnesota Satisfaction Questionnaire^37^, results based on 4 participants from the intervention group and 5 from the control group (those employed or on sick leave at the time of the study).

^b^For the newly added outcomes, 15 and 13 participants provided data from the intervention and control group respectively.

### Participants’ adherence to intervention

A total of 14 participants completed all 18 training sessions as planned (40% compliance) whereas 17 completed more than 50% of sessions (range 10–17), and 4 less than 50% (range 2–7). Considering that the intervention period was shortened for some participants due to the variable rate of surgical operation for elective surgeries, we can consider that a maximum of 569 sessions could be provided to participants yielding a compliance rate of 90.3% (288/569) with a mean of 14.7 sessions provided per participant. Assessment of physical activities performed outside of the study protocol at the post-intervention assessment was similar in both groups (p = 0.39) with 8 and 10 participants reporting being active in the intervention and control group respectively. (Results based on 29 individuals in the intervention group and 26 in the control group). Types of physical activity included treadmill or outdoor walking, stationary or outdoor cycling, snowshoeing, fall risk prevention program, and performing the prehabilitation exercises on off days.

### Participants’ adherence to surgical plan

Out of the 68 enrolled participants, 4 did not undergo surgery as planned. All 3 individuals from the control group opted out because the risks associated with the surgery were perceived as too high given their advanced age or concomitant health issues. The one individual that opted out of surgery in the intervention group did so in accordance with the neurosurgeon’s opinion that her functional status had improved beyond surgical candidacy. In total 64 participants underwent lumbar laminectomy/laminotomy over the course of the study and none underwent a revision surgery within the 6-month follow-up.

### Outcomes and estimation of intervention effect

#### Results of analyses conducted using preoperative data

**Figure 2:**
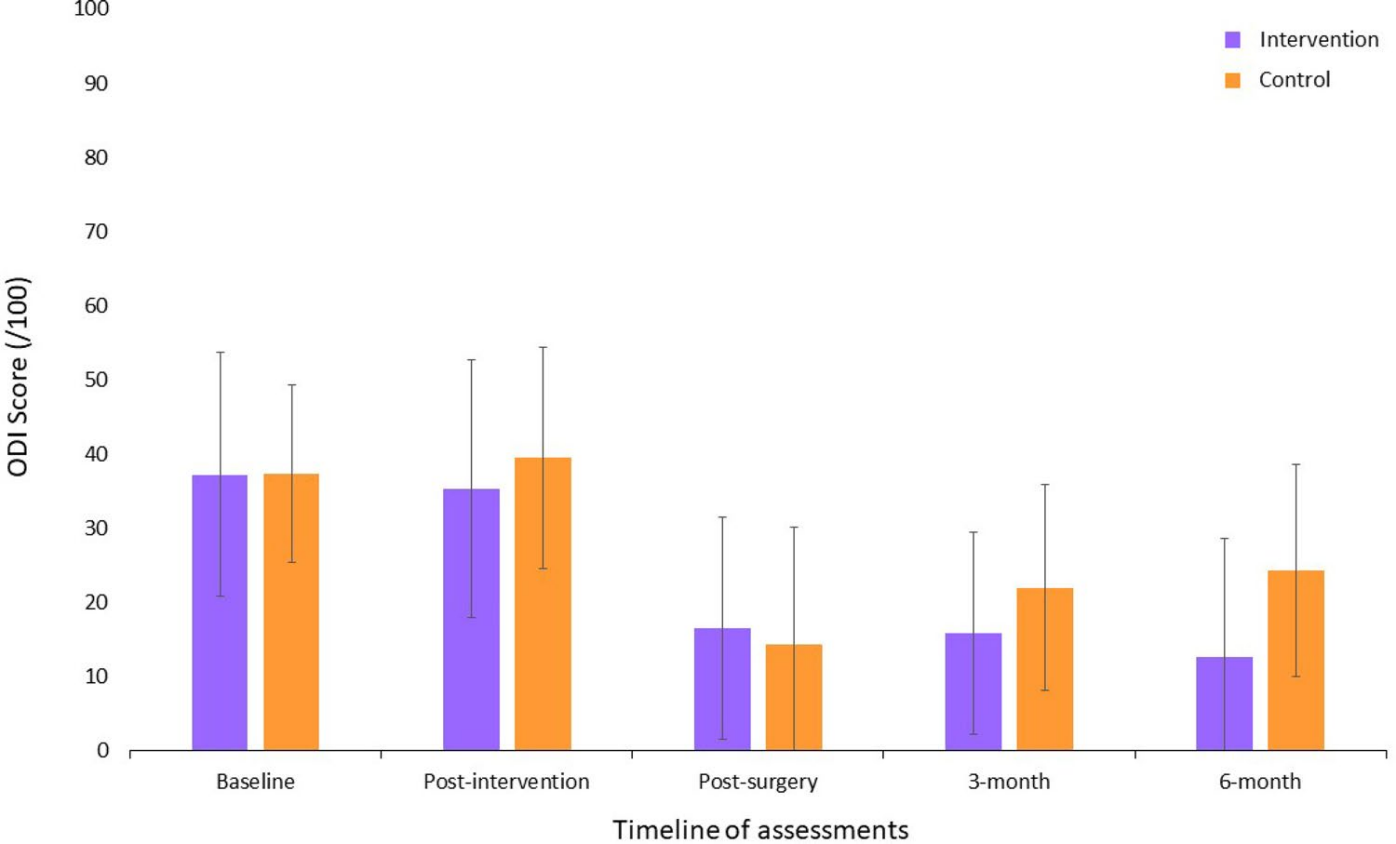
Comparison of low back related disability (means ± SD) (Greater scores indicate greater disability; *ODI* Oswestry disability index).

**Figure 3:**
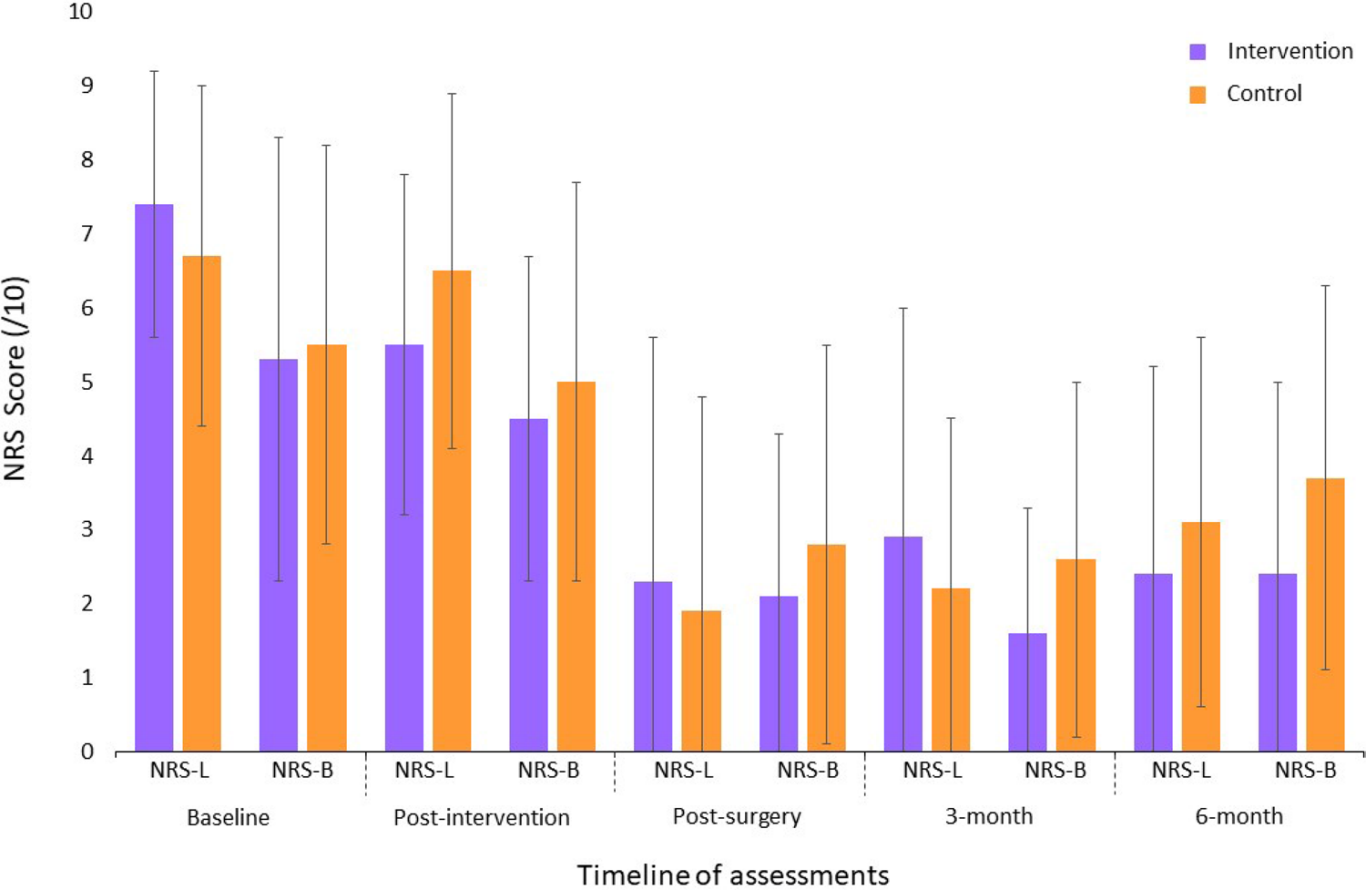
Comparison of leg and back pain intensity (means ± SD) (Greater scores indicate greater pain intensity; *NRS-L* numerical rating scale for leg pain intensity, *NRS-B* numerical rating scale for low back pain intensity).

**Figure 4:**
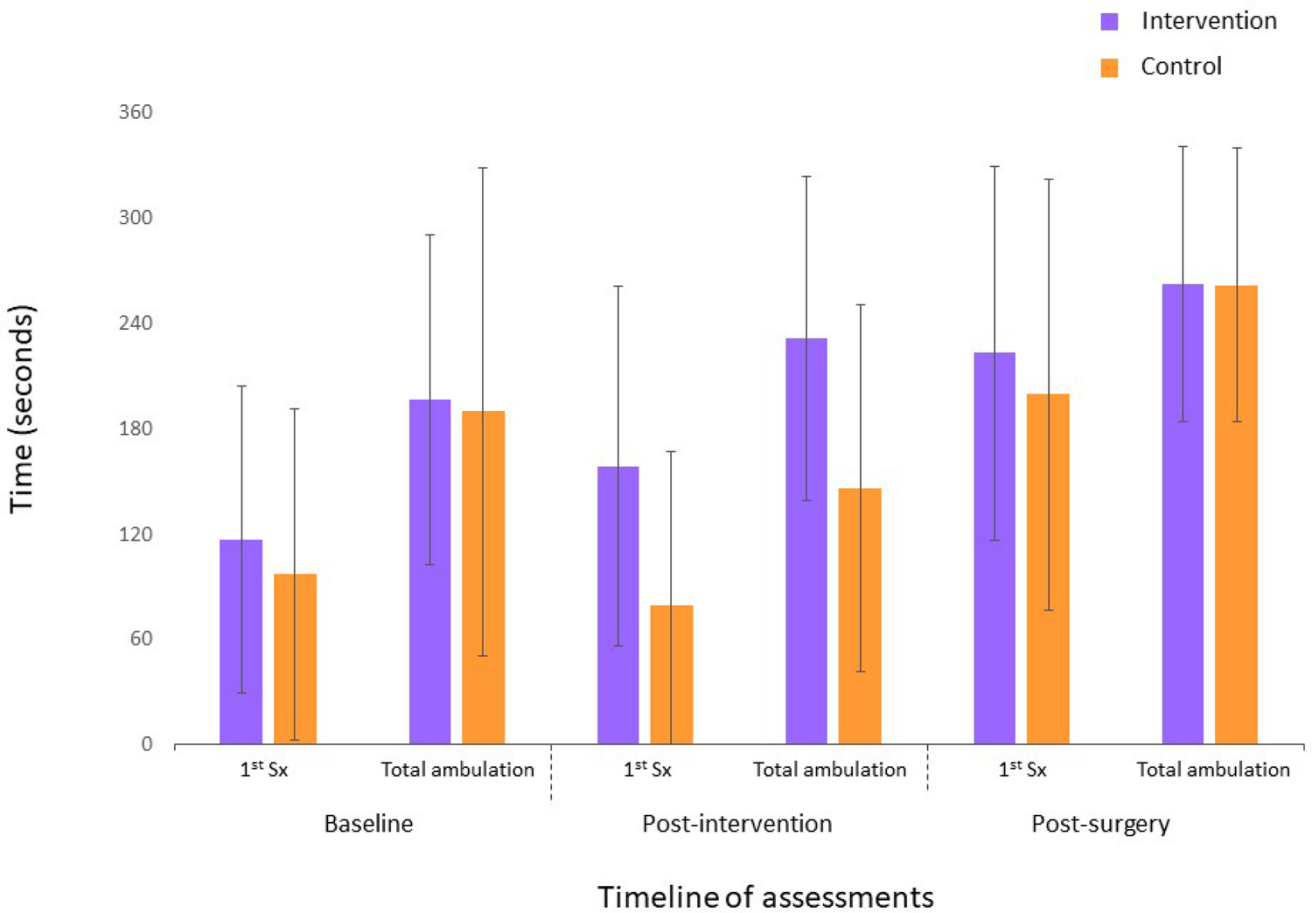
Comparison of walking capacities (means ± SD) (Greater scores indicate better walking capacities; *1st Sx* walking time to first symptoms).

Significant Group × Time interactions were found between the baseline and the preoperative assessments in favor of the intervention group for leg pain intensity (F_1,66_ = 4.63, p = 0.03, ηp^2^ = 0.07); LSS-related disability (F_1,25_ = 4.48, p = 0.04, ηp^2^ = 0.15); maximum lumbar strength in flexion (F_1,66_ = 5.15, p = 0.02, ηp^2^ = 0.07); low back extensor muscles endurance (F_1,63_ = 6.67, p = 0.002, ηp^2^ = 0.15); total ambulation time (F_1,64_ = 5.63, p = 0.02, ηp^2^ = 0.08); and sit to stand (F_1,25_ = 7.02, p = 0.01, ηp^2^ = 0.22). Figures 2, 3 and 4 present between group comparisons of low back related disability, leg and back pain intensity and walking capacities respectively.

#### Results of analyses conducted using postoperative data

The only significant Group × Time interaction found in the postoperative period, was for low back-related disability in favor of the intervention group (F_2,132_ = 6.20, p = 0.003, ηp^2^ = 0.06) with the largest difference being at 6 months. Means, standard deviations and 95% confidence intervals for each group are presented based on clinical and physical outcomes for all time points in Tables 2 and 3 respectively.

**Table 2:** Results for clinical outcome measures.

Variable	N	Intervention group	N	Control group	Main effect of time (*p*)	Main effect of group (*p*)	Group × time interaction (*p*)
		Mean + SD (95% CI)		Mean + SD (95% CI)			
**Back pain intensity/10**							
Baseline	35	5.4 ± 3.0 (4.3 to 6.4)	33	5.6 ± 2.7 (4.6 to 6.5)	0.09	0.69	0.91
Post-intervention	29	4.5 ± 2.2 (3.6 to 5.3)	26	5.0 ± 2.7 (3.9 to 6.1)			
Post-surgery	32	2.1 ± 2.2 (1.3 to 2.9)	21	2.8 ± 2.7 (1.5 to 3.9)	0.01	0.17	0.90
3 months	24	1.6 ± 1.7 (0.9 to 2.3)	23	2.6 ± 2.4 (1.5 to 3.6)			
6 months	24	2.4 ± 2.6 (1.3 to 3.5)	19	3.7 ± 2.6 (2.4 to 4.9)			
**Leg pain intensity/10**							
Baseline	35	7.4 ± 1.9 (6.8 to 8.1)	33	6.7 ± 2.4 (5.9 to7.6)	0.001	0.76	**0.03**
Post-intervention	29	**5.5 ± 2.3 (4.6 to 6.4)**	26	6.5 ± 2.4 (5.5 to 7.4)			
Post-surgery	32	2.3 ± 3.3 (1.1 to 3.5)	21	1.9 ± 2.9 (0.6 to 3.2)	0.11	0.99	0.30
3 months	24	2.9 ± 3.1 (1.6 to 4.3)	23	2.2 ± 2.3 (1.2 to 3.2)			
6 months	24	2.4 ± 2.8 (1.2 to 3.6)	19	3.1 ± 2.5 (1.8 to 4.3)			
LSS disability/55 or 79*							
Baseline	15	39.5 ± 4.3 (36.6 to 42.4)	13	38.0 ± 4.2 (35.5 to 40.6)	0.005	0.47	**0.04**
Post-intervention	9	**35.0 ± 7.9 (28.9 to 41.0)**	8	37.5 ± 6.5 (32.1 to 42.9)			
Post-surgery*	13	29.6 ± 8.5 (24.5 to 34.7)	6	28.5 ± 10.6 (17.4 to 39.6)	0.27	0.72	0.83
3 months*	8	37.9 ± 10.2 (29.3 to 46.4)	6	39.2 ± 14.4 (24.0 to 54.3)			
6 months*	9	30.5 ± 10.1 (22.7 to 38.4)	3	45.0 ± 21.6 (8.8 to 78.8)			
**Back disability/100**							
Baseline	35	37.2 ± 16.5 (32.5 to 42.9)	33	37.5 ± 11.9 (33.2 to 41.7)	0.95	0.29	0.12
Post-intervention	29	35.3 ± 17.4 (28.7 to 41.9)	26	39.5 ± 14.9 (33.5 to 45.5)			
Post-surgery	32	16.5 ± 15.0 (11.0 to 22.0)	21	14.3 ± 15.8 (7.1 to 21.5)	0.02	0.36	**0.003**
3 months	24	15.8 ± 13.6 (10.1 to 21.6)	23	**22.0 ± 13.9 (15.9 to 28.0)**			
6 months	24	12.7 ± 16.0 (6.1 to 19.4)	19	**24.3 ± 14.3 (17.6 to 31.0)**			
**Kinesiophobia/68**							
Baseline	35	47.5 ± 8.1 (44.7 to 50.3)	33	45.2 ± 7.7 (42.5 to 47.9)	0.74	0.90	**0.02**^**ǂ**^
Post-intervention	29	44.2 ± 7.7 (41.3 to 47.1)	26	47.6 ± 8.0 (44.4 to 50.8)			
Post-surgery	32	38.2 ± 8.9 (35.0 to 41.4)	21	38.9 ± 9.3 (34.7 to 43.2)	0.47	0.48	0.78
3 months	24	37.9 ± 7.8 (34.7 to 41.1)	23	41.3 ± 7.3 (38.1 to 44.5)			
6 months	24	37.6 ± 7.6 (34.5 to 40.7)	19	40.4 ± 10.7 (35.5 to 45.3)			
**Depression/63**							
Baseline	35	4.4 ± 5.1 (2.7 to 6.2)	33	4.7 ± 4.2 (3.2 to 6.2)	0.42	0.28	0.97
Post-intervention	29	4.3 ± 5.3 (2.3 to 6.3)	26	5.3 ± 5.2 (3.2 to 7.3)			
Post-surgery	32	2.0 ± 3.4 (0.8 to 3.3)	21	1.8 ± 3.1 (0.4 to 3.3)	0.009	0.87	0.87
3 months	24	2.7 ± 3.5 (1.3 to 4.2)	23	3.9 ± 4.6 (1.9 to 5.9)			
6 months	24	1.7 ± 2.0 (0.8 to 2.5)	19	3.4 ± 3.3 (1.9 to 4.9)			

*SD* standard deviation, *CI* confidence interval.

*LSS-related disability measured using the Swiss Spinal Stenosis Questionnaire—maximum score at baseline and post-intervention assessments is 55 (includes symptoms and function subscales) and maximum score at postoperative, 3-month and 6-month assessments is 79 (includes symptoms, function and satisfaction subscales).

^ǂ^The Group × Time interaction term was statistically significant but post-hoc analysis using Bonferroni test revealed no significant within or between group differences.

Bold indicate a significant intra-group change from baseline (for analyses conducted between baseline and post-intervention assessments) or intra-group change from post-surgery (for analyses conducted between post-surgery and follow-ups assessments).

**Table 3:** Results for physical outcome measures.

Variable	N	Intervention group	N	Control group	Main effect of time (*p*)	Main effect of group (*p*)	Group × time interaction (*p*)
		Mean + SD (95% CI)		Mean + SD (95% CI)			
**Trunk muscles strength N m**							
Flexion							
Baseline	35	46.8 ± 23.4 (38.7 to 54.9)	33	44.1 ± 28.1 (33.7 to 54.3)	0.02	0.16	**0.03**
Post-intervention	29	**53.2 ± 24.8 (43.4 to 63.1)**	26	42.1 ± 26.1 (30.5 to 53.7)			
Post-surgery	32	54.4 ± 28.5 (43.1 to 65.7)	21	50.1 ± 27.8 (37.9 to 63.9)	0.32		
Extension							
Baseline	35	32.8 ± 29.2 (22.8 to 42.9)	33	26.7 ± 26.3 (17.2 to 36.2)	0.004	0.21	0.51
Post-intervention	29	57.0 ± 61.0 (32.4 to 81.6)	26	26.3 ± 26.7 (14.1 to 38.5)			
Post-surgery	32	46.4 ± 41.3 (29.8 to 63.1)	21	48.5 ± 40.0 (29.2 to 67.8)	0.85		
**Lumbar active ROMs (degrees)**							
Flexion							
Baseline	35	66. 0 ± 25.2 (56.9 to 75.1)	33	67.2 ± 20.9 (59.1 to 75.3)	0.69	0.72	0.15
Post-intervention	29	68.6 ± 27.2 (57.6 to 79.6)	26	62.3 ± 25.8 (50.6 to 74.0)			
Post-surgery	32	68.8 ± 17.6 (61.9 to 75.8)	21	72.9 ± 19.4 (63.6 to 82.3)	0.45		
Extension							
Baseline	35	14.4 ± 7.1 (11.7 to 17.2)	33	14.4 ± 5.5 (12.0 to 16.8)	0.28	0.10	0.05
Post-intervention	29	16.8 ± 6.9 (13.9 to 19.7)	26	12.7 ± 7.1 (9.4 to 15.9)			
Post-surgery	32	17.7 ± 6.7 (15.1 to 20.4)	21	15.2 ± 5.9 (12.4 to 18.0)	0.15		
Left lateral flexion							
Baseline	35	13.0 ± 7.3 (10.4 to 15.7)	33	12.1 ± 6.2 (9.7 to 14.5)	0.97	0.30	0.37
Post-intervention	29	14.1 ± 8.8 (10.6 to 17.6)	26	10.6 ± 5.3 (8.2 to 13.0)			
Post-surgery	32	13.5 ± 5.7 (11.3 to 15.8)	21	12.8 ± 5.4 (10.2 to 15.4)	0.63		
Right lateral flexion							
Baseline	35	13.8 ± 7.9 (10.9 to 16.7)	33	13.8 ± 6.8 (11.1 to 16.4)	0.26	0.65	0.29
Post-intervention	29	13.2 ± 8.2 (9.9 to 16.4)	26	11.2 ± 5.5 (8.7 to 13.7)			
Post-surgery	32	14.4 ± 6.6 (11.8 to 17.1)	21	15.3 ± 7.9 (11.5 to 19.1)	0.75		
**Right knee extensors strength—lbs**							
Baseline	35	62.2 ± 31.7 (51.3 to 73.1)	33	60.5 ± 28.7 (50.6 to 70.9)	0.45	0.20	0.54
Post-intervention	29	65.6 ± 29.9 (53.5 to 77.7)	26	54.2 ± 31.5 (41.2 to 67.2)			
Post-surgery	32	75.7 ± 37.6 (61.7 to 89.8)	21	62.4 ± 28.3 (49.5 to 75.3)	0.49		
**Left knee extensors strength—lbs**							
Baseline	35	72.6 ± 27.3 (55.3 to 89.9)	33	75.6 ± 26.9 (60.1 to 91.2)	0.45	0.54	0.20
Post-intervention	29	75.4 ± 26.2 (55.3 to 95.5)	26	69.3 ± 31.9 (46.5 to 92.1)			
Post-surgery	32	87.5 ± 24.4 (72.8 to 102.3)	21	80.7 ± 27.4 (55.3 to 106.0)	0.30		
**Lumbar extensors endurance—s**							
Baseline	32	45.2 ± 59.9 (23.6 to 66.8)	30	38.8 ± 49.2 (20.4 to 57.1)	0.61	0.14	**0.002**
Post-intervention	27	65.7 ± 65.6 (39.8 to 91.7)*	23	17.9 ± 35.8 (2.4 to 33.4)*			
Post-surgery	30	69.5 ± 59.2 (47.4 to 91.6)	21	57.3 ± 60.5 (29.8 to 84.8)	0.88		
**Walking capacities—s**							
Time to 1st symptoms							
Baseline	35	116.8 ± 87.4 (86.8 to 146.8)	33	97.4 ± 93.4 (63.1 to 131.7)	0.44	0.01	0.08
Post-intervention	29	158.8 ± 102.4 (119.8 to 197.7)	25	79.4 ± 87.4 (43.3 to 115.4)			
Post-surgery	30	223.1 ± 106.5 (182.6 to 263.6)	21	199.6 ± 122.8 (143.7 to 255.5)	0.39		
Total ambulation time							
Baseline	35	196.8 ± 94.0 (163.9 to 229.6)	33	190.1 ± 139.1 (139.9 to 240.2)	0.81	0.03	**0.02**
Post-intervention	29	231.7 ± 92.2 (195.9 to 267.5)*	25	146.3 ± 104.7 (103.1 to 189.5)*			
Post-surgery	30	262.2 ± 78.5 (232.3 to 292.0)	21	262.0 ± 78.1 (226.5 to 297.6)	0.85		
**Get up and go—s**							
Baseline	15	8.8 ± 1.7 (7.7 to 9.9)	13	10.2 ± 4.1 (7.7 to 12.7)	0.007	0.15	0.12
Post-intervention	9	7.5 ± 1.9 (6.0 to 8.9)	8	9.7 ± 2.6 (7.7 to 11.7)			
Post-surgery	13	6.7 ± 1.3 (5.9 to 7.5)	6	8.0 ± 2.7 (5.2 to 10.9)	0.09		
**Sit to stand—repetitions**							
Baseline	15	9.7 ± 3.8 (7.2 to 12.1)	13	9.1 ± 2.7 (7.4 to 10.7)	0.001	0.14	**0.01**
Post-intervention	9	**12.1 ± 3.6 (9.4 to 14.9)**	8	8.4 ± 2.2 (6.8 to 10.1)			
Post-surgery	13	11.8 ± 2.2 (10.4 to 13.1)	6	12.0 ± 2.1 (9.8 to 14.2)	0.89		

*SD* standard deviation, *CI* confidence interval, *ROM* ranges of motion.

Bold indicate a significant intra-group change from baseline.

*Denotes a significant between group difference.

### Quality of life

Changes in quality of life were assessed by comparing the proportions of individuals from each group that reported an improvement in each of the questionnaire 5 dimensions. No between group significant difference was found at any of the assessment timepoints (all ps > 0.05).

### Participants’ perceived change in global status

At the preoperative assessment, participants in the intervention group reported greater positive change in their global status (mean ± SD: 2.9 ± 1.3) compared to the control group (4.5 ± 1.0). Sixty-nine per cents reported that their status had “improved” (= very much better, much better, or slightly better) in the intervention group compared to 11.5% in the control group (p < 0.001). Similarly, 13% percent reported that their status had “worsened” (= very much worse, much worse, or slightly worse) in the intervention group compared with 46% in the control group (p < 0.01).

### Participants’ satisfaction

Thirty-one participants provided an objective evaluation of their overall satisfaction with the intervention program. The mean satisfaction score (mean ± SD) reached 94.4% ± 8.3. Both groups provided similar satisfaction rates with regards to postoperative back pain outcome with a score (mean ± SD) of 84.4% ± 22.2 for the intervention group and of 84.0% ± 22.5 for the control group (p = 0.23). Satisfaction rates for postoperative leg pain outcome were also similar with 82.3% ± 23.4 in the intervention group and 84.6% ± 23.9 in the control group (p = 0.34).

### Intraoperative data

Intraoperative measures and length of hospital stay were similar in both groups. Results are presented in Table 4.

**Table 4:** Perioperative data.

	Intervention (n = 34) Mean ± SD	Control (n = 30) Mean ± SD	*p*
Length of surgery (min)	101.3 ± 47.9	105.2 ± 57.8	0.77
Blood loss (ml)	213.4 ± 243.1	221.0 ± 226.3	0.90
Intraoperative complication (n)	0	2	–
Length of hospital stay (days)	4.1 ± 3.2	4.5 ± 2.0	0.58
Minimally invasive surgey (n)	11	8	0.62
Open surgery (n)	23	22	0.27
Received physiotherapy postoperatively (n)	3	3	0.87

Physiotherapy consisted of one hospital-based or home-based visit to ensure adequate independency. The intraoperative complications were dural tears.

### Harms

At no point in time were adverse events reported as a result of either the training program or physical assessments.

## Discussion

The aim of the present study was to assess the effectiveness of an exercise-based prehabilitation program, compared to usual care, on improving preoperative capacities, and postoperative recovery in patients with lumbar spinal stenosis. The results showed improvements in both self-reported clinical and objective physical outcomes at the post-intervention assessment in favor of the prehabilitation group. However, theses differences were not maintained after the surgery. As such, in the postoperative phase, back-related disability was the only parameter that followed distinct trajectories between groups, with improvements seen in the intervention group and deteriorations seen in the control group, over the 6-month follow-up.

### Clinical significance

The within group differences observed in the intervention group after the prehabilitation intervention were clinically significant for decreased leg pain intensity^38^ (− 1.9 point), LSS-related disability^38^ (− 4.5 points), and the sit-to-stand test^39^ (+ 2.4 repetitions) (major clinically important improvement determined from patients with hip osteoarthritis ≥ 2). To the best of our knowledge no minimal clinically important difference has been determined for maximum isometric flexor strength and trunk extensor endurance. We noted a 13.7% increase in trunk flexor strength (+ 6.4 Nm) and a 45% improvement in low back extensor endurance (+ 20.5 s) from baseline. On the other hand, improvement in total ambulation time (+ 34.9 s = 17.7%) did not reach the proposed 30% threshold for clinical significance^40^.

The between group differences identified for low back extensor endurance (+ 47.8 s = 267.0% increase) and total ambulation time (+ 85.4 s = 58.4% increase) correspond to a large and medium effect size, respectively^41^.

### Comparison with other trials

As of today, only few publications have investigated the effectiveness of prehabilitation interventions within the context of spine surgery. In the systematic review published in early 2021 by Janssen et al. a total of 15 studies were included, most of which (13/15) investigated cognitive behavioral therapy. The authors concluded based on meta-analyses that there was very low to moderate quality evidence that prehabilitation has no effect compared to usual care on physical functioning, leg and back pain intensity, health-related quality of life, depression, anxiety, length of hospital stay, and analgesics use. The present trial adds on to the review of Janssen et al. by being the first to report on adverse event related to both the prehabilitation intervention and physical assessments. In addition, we did not exclude patients with multiple comorbidities or with previous history of surgery and imposed no maximum age limit, which were identified as limitations in previous studies.

Results of the present study somewhat contrast with the results previously reported by Nielsen et al.^16^ which included greater function prior to surgery, and faster postoperative recovery and discharge from hospital in favor of the intervention group compared to the standard care group. Their proposed intervention combined 6 to 8 weeks of preoperative daily individualized home training program, preoperative supplemental food intake, and early in-hospital postoperative rehabilitation. In comparison, we decided to include only one aspect of the recommended prehabilitation triad (exercise training, nutrition, and emotional wellbeing)^6^ in order to tease out the effects of exercises alone given that evidence on how to best prepare for spine surgery is scarce. However, there is evidence from studies investigating major surgeries that supports the use of multimodal interventions, including modification of behavioural and lifestyle risk factors, to improve surgical outcome rather than solely focusing on the underlying disease process^42^. In addition, despite the fact that the focus of prehabilitation has primarily been put on the optimisation of physical comorbidities, there is an increasing body of evidence that emphasizes the role of preoperative psychological factors^43^ on both physical and psychological postoperative outcomes. Likewise, patients with high-risks profiles, such as frailty and comorbidity, have been proposed to be the ones that would most benefit from prehabilitation^44–46^. Thus, pre-operative risk stratification taking into account both modifiable and non-modifiable risk factors of poor surgical outcome and complications would allow to tailor prehabilitation interventions to the patients’ needs and capacities^42,47^. Nevertheless, such endeavour to optimize patients’ preoperative status requires multidisciplinary input and substantial resources to ensure proper monitoring.

### Strengths and limitations

Considering the limited available evidence on the effect of prehabilitation interventions within the context of spine surgery, the results of the present study should be interpreted with caution. Among its strengths, the study followed a randomised, controlled design and complied with the related Consolidated Standards of Reporting Trials guideline. Furthermore, the proposed intervention could be suited to individual participants’ level of physical capacities and no substantial protocol modifications were required to accommodate day-to-day variation in patients’ symptomatology. The intervention was also delivered by a single certified kinesiologist to decrease the probability of a clinician effect and avoid inter-clinician variations. Of importance, the study reflects a pragmatic approach to rehabilitation intervention within the Quebec public health care system, characterized by the variable intervention length based on the surgical waitlist. Participating individuals came from both Trois-Rivières and its surrounding areas, which allows to infer the results to other Canadian provinces with comparable public health care system. However, the results cannot be extrapolated to patients with spinal instability or primary foraminal stenosis. Based on the collaborating neurosurgeons’ experience, patients requiring fusion surgery (instrumented or not) or foraminotomy would undergo more complex procedures and have different recovery pathways and were therefore not included in the study. With regards to the methods, missing data were dealt with using multiple imputations so that reasonable power could be maintained when conducting the statistical analyses.

In contrast, the study also has limitations, of which the first one is the use of the pilot study data. Although using data from pilot study to conduct sample size estimate is widely debated in the literature, there was no pooled results available at the time the study was conducted^48^. In addition, estimating variance from a small pool of data may inflate the type I error rate when conducting the main study^49^. Also, it will not be possible in future meta-analysis to use the data from both the pilot study and the main trial because the data of pilot study have been included in the main study and are no longer independent.

Similarly, the fact that the sample size fell short of the targeted number at enrollment combined with a high drop out rate increased data heterogeneity which limits the power for some of the analyses. On the one hand, recruitment was hampered by self-perceived health-related barriers and transportation issues. To have provided education at the time of recruitment with regards to the benefits of being active, beyond study purposes, could have improved participation rate^47^. Likewise, to have offered the option to perform the exercise program at home, in the instance where it could be done safely without supervision, could have facilitated recruitment^50^. On the other hand, the decision to prematurely stopped recruitment due to unforeseen organizational and time constraints at the local hospital which significantly limited the referral pathway from the neurosurgery unit also played a role in having a small final sample size.

With regards to limitations in the conduct of the study, the principal investigator was not blind to participants’ group allocation while conducting the assessments, which may have led to measurement bias. Finally, considering that adding outcomes after the pilot phase of the study resulted in fewer than half of the participants providing data (Swiss Spinal Stenosis questionnaire, get up and go test, and sit to stand test) which greatly limit their interpretation, these results should be viewed as preliminary.

Overall, the current body of evidence on the effectiveness of prehabilitation program designed for spine surgery is less robust than that of other surgical contexts and appears to be less promising. Considering that patients awaiting elective surgery may have longer preoperative windows and, for some, lesser complex clinical profiles than those awaiting non-elective surgery, preoperative interventions may play a different role than augmenting fitness for surgery. As such, many of the participants in the present study did not seek conservative care prior to undergoing surgery. Given that for patients with stable clinical status, a trial of conservative care is recommended prior to surgical management^51^, preoperative interventions may be beneficial in terms of clinical improvements and allow to better detect those for whom surgery is necessary to regain satisfactory functional capacities.

## Conclusion

The main objective of the present study was to evaluate the effectiveness of a 6-week preoperative exercise-based program, compared to usual care, in patients awaiting elective surgery for LSS. Our findings suggest that while the intervention yielded improvements on clinical status and physical capacities preoperatively, it was insufficient to foster a more rapid shot-term postoperative recovery. Tailored prehabilitation based on stratification of high-risk patient profiles coupled with education should make the object of future studies looking at preoperative intervention in the context of spinal surgery.
